# Multimodal Emotion Evaluation: A Physiological Model for Cost-Effective Emotion Classification

**DOI:** 10.3390/s20123510

**Published:** 2020-06-21

**Authors:** Gisela Pinto, João M. Carvalho, Filipa Barros, Sandra C. Soares, Armando J. Pinho, Susana Brás

**Affiliations:** 1 Department of Electronics, Telecommunications and Informatics, University of Aveiro, 3810-193 Aveiro, Portugal; gisela.pinto@ua.pt (G.P.); joao.carvalho@ua.pt (J.M.C.); ap@ua.pt (A.J.P.); 2 Institute of Electronics and Informatics Engineering of Aveiro (IEETA), 3810-193 Aveiro, Portugal; 3 Department of Education and Psychology, University of Aveiro, 3810-193 Aveiro, Portugal; fmbarros@ua.pt (F.B.); sandra.soares@ua.pt (S.C.S.); 4 William James Center for Research, University of Aveiro, 3810-193 Aveiro, Portugal; 5 Center for Health Technology and Services Research, University of Aveiro, 3810-193 Aveiro, Portugal

**Keywords:** affective computing, multimodal, feature extraction, random forest, neural network

## Abstract

Emotional responses are associated with distinct body alterations and are crucial to foster adaptive responses, well-being, and survival. Emotion identification may improve peoples’ emotion regulation strategies and interaction with multiple life contexts. Several studies have investigated emotion classification systems, but most of them are based on the analysis of only one, a few, or isolated physiological signals. Understanding how informative the individual signals are and how their combination works would allow to develop more cost-effective, informative, and objective systems for emotion detection, processing, and interpretation. In the present work, electrocardiogram, electromyogram, and electrodermal activity were processed in order to find a physiological model of emotions. Both a unimodal and a multimodal approach were used to analyze what signal, or combination of signals, may better describe an emotional response, using a sample of 55 healthy subjects. The method was divided in: (1) signal preprocessing; (2) feature extraction; (3) classification using random forest and neural networks. Results suggest that the electrocardiogram (ECG) signal is the most effective for emotion classification. Yet, the combination of all signals provides the best emotion identification performance, with all signals providing crucial information for the system. This physiological model of emotions has important research and clinical implications, by providing valuable information about the value and weight of physiological signals for emotional classification, which can critically drive effective evaluation, monitoring and intervention, regarding emotional processing and regulation, considering multiple contexts.

## 1. Introduction

Emotions are adaptive responses to environmental stimuli, involving alterations in subjective experience, as well as in cognitive, motivational, physiological, and behavioral domains [1]. These responses interact strongly with decision-making, perception, and learning [2] and play a pivotal role in survival and well-being, by allowing the necessary resources to deal with everyday opportunities and threats [3]. Every emotion comprises a functional role and empowers people with the ability to respond to challenging situations.

Notwithstanding the adaptive value of emotions, when persistent and/or exaggerated, they may be associated with significant discomfort and suffering, with deficits in multiple areas of functioning, as well as with the development of physical and mental health problems, such as cardiovascular and anxiety disorders [4,5]. Therefore, the ability to correctly detect, recognize, and regulate emotions may be preponderant for how people interact with their multiple life contexts and, ultimately, for their well-being and quality of life.

Knowledge about emotional perception and response is a crucial tool in this process, by allowing the understanding of peoples’ preferences, strategies, and abilities to adapt and respond across contexts. Furthermore, detecting, processing, and interpreting emotional response using physiological data acquisition and processing systems may constitute an objective, safe, and efficient way to gather and use emotional information for these purposes [6]. 

The process of emotion identification allows a better interaction with computers, systems, and environments, enabling an improved user experience [7,8]. In fact, there is a plethora of opportunities where emotion recognition would be beneficial [7,8,9], from computer interaction to mental health issues (e.g., to provide emotional response monitoring during therapy). Emotions regulate body changes through activation of the central and peripheral nervous system, being this translated as behavioral (facial expression, speech, posture) and/or physiological responses (electroencephalogram (EEG), electrocardiogram (ECG), electromyogram (EMG)) [6,10]. For instance, in the presence of emotional triggers, our heart activity changes, our facial expression is different, and our muscles compress [11]. So, emotional responses may be described by the quantification of body information [10,11].

The literature usually presents emotion identification systems based on one signal [6,9,10,12,13]. Notwithstanding, it is known that some signals present a premature response to emotion in comparison with others [11]. Therefore, a system able to combine information from different sources could be more efficient.

Several researchers explored the combination of information to identify emotions. For instance, Jing Cai and colleagues [14] used a multichannel physical signal recording instrument, namely Biopac System MP150, to record the ECG signal. During the recording time, participants watched 20–30 min film clips, to allow the induction of Joy and Sadness emotions. They used the Tobu Search Algorithm to perform feature selection. Additionally, Fisher k nearest neighbors (Fisher-KNN) was used for emotion classification. For Joy, an average of 81.29% was obtained; for sadness, the average recognition was 90.63%.

In another study, Jordan Bird and colleagues [15] used EEG brainwave data to classify emotional experiences. Positive and Negative emotional states were elicited using film clips. For classification, random forest was used. An overall accuracy of around 97.89% was obtained.

Rigas and colleagues [16] analyzed physiological signal to recognize three emotional classes: Happiness, Disgust and Fear, using a dataset of nine healthy subjects. The physiological signals used were electromyogram (EMG), electrocardiogram (ECG), respiration (RSP), and electrodermal activity (EDA). The approach consisted of four steps: biosignal acquisition, biosignal preprocessing and feature extraction, feature selection, and classification. For feature extraction, Simba and Principal Component Analysis (PCA) algorithm was used. The methods used to classify were k-nearest neighbor and random forest. Using Simba feature selection, the best performance was obtained when used with k-nearest neighbor, which was around 62.70%. With the PCA approach, the best accuracy was obtained using Random Forest and consisted of around 50.81%.

Mokhayeri and colleagues [17] used emotional identification systems to detect mental stress and relaxation. The physiological signals used were pupil diameter (PD), electrocardiogram (ECG), and photoplethysmogram (PPG). The most relevant features were extracted from each signal, and the optimized features were selected by using the genetic algorithm and imported into the Fuzzy Support Vector Machine (SVM) to classify stress and relaxation states. The accuracy obtained was 78.5%.

Khadidja Gouizi [18] analyzed six basic emotions: neutrality, joy, fear, sadness, disgust, and amusement. These emotions were induced using the international affection picture system (IAPS). The physiological signals analyzed were electromyogram (EMG), respiratory volume (RV), skin temperature (SKT), skin conductance (SKC), blood volume pulse (BVP), and heart rate (HR). The classification algorithm used was the support vector machine, resulting in a recognition rate of 85% for different emotional states.

Several studies have investigated emotion classification systems, but most of them are based on the analysis of only one or a few physiological signals. Knowing how informative the individual signals are and how their combination works, would allow to develop more cost-effective, informative, and objective systems for emotion detection, processing, and interpretation.

In the present work, our aims are twofold: (i) evaluate which signal/feature combination better describes an emotion; (ii) analyze which isolated signal may better describe an emotion. To accomplish these goals, data were collected from volunteers that watched emotional videos eliciting happiness, fear and nothing in particular (neutral emotion condition), while their physiological signals (ECG, EDA, EMG zygomatic and medial frontal) were recorded. The most informative five-minute window was selected by means of information quantity. Classifiers were designed considering a multimodal approach, and different signal’s combinations were tested and evaluated. Therefore, this work aims to present a modular emotion identification system that may be used in emotion stratification and description, which can operate independently of the signal data source (between the ones available in the work design). 

The development of automatic systems for emotion identification and modelling has the potential to greatly support research and practice in areas where emotional data can be preponderant to effectively predict individual’s emotion, cognition and behavior, such as in mental health settings. In fact, the Global Burden of Diseases, Injuries, and Risk Factors Study of 2017 [19] indicates that some mental disorders, such as depressive and anxiety disorders, are part of the leading causes of an elevated number of years lived with disability. Nowadays, this yields even more relevance considering the new challenges associated with the COVID-19 pandemic, including the increase of anxiety, stress and depression rates at a global level [20,21]. Having access to complementary information of this nature (physiological activation) would greatly enrich the process of monitoring and intervention in these situations and could even foster the development and improvement of prevention programs.

Grounded on these premises, the present work is organized as follows: Section 2 describes the experiment and the methods used; Section 3 explains the results obtained, which are, lastly, discussed in Section 4.

## 2. Materials and Methods

In order to study the physiological component of emotions, a carefully designed protocol was implemented to observe and collect the physiological responses of volunteers to video stimuli. 

### 2.1. The Physiological Database

All study procedures were according to the guidelines of the Declaration of Helsinki and the American Psychological Association. It was also submitted and approved by the Ethics and Deontology Council of the University of Aveiro, Portugal. Before the experiment began, all necessary information was provided to the participants, describing the study, and informing that they can withdraw the study at any time. An informed consent was signed, assuring that the participant participates in the study voluntarily and understand all study procedure.

This study intends to evaluate the response to happiness, fear and neutral emotional conditions. To accomplish such goal, the participants performed three sessions, spaced for at least one week. This temporal separation avoids emotional contagion between sessions. The exposition order to emotions were counterbalanced. The participant was instructed to seat comfortably, to be as attentive as possible to the task, and place his/her chin on a chin rest, in front of a computer. Before and after the emotional induction, participants evaluate their emotional state in terms of happy, fear, anxiety, and stress, by four Visual Analogue Scales (VAS) of 100 points.

In order to evaluate the physiological response to emotions, a visual elicitation emotional video was displayed. This video was preceded by a documentary excerpt of about 5 min; this was considered the baseline, since no emotion was induced. Emotional elicitation was performed by three sets of 8–12 movie excerpts, completing a 30 min duration film presentation. Fear was induced by excerpts of terror movies. Happiness was induced by excerpts of comedy movies. The neutral condition was induced by documentary excerpts (please refer to the work of Ferreira and colleagues [12] for a similar procedure). The emotional content of the excerpts used on video display were evaluated on a previous pilot study. In this pilot study, several movie excerpts were presented to participants and they ranked their emotional levels, which allowed to infer the effectiveness of the excerpts in inducing happiness, fear, and neutral emotions.

Fifty-five volunteers participated in this study, 18 males and 37 females, aged between 18 and 28 years old (21 ± 2.62). Following the inclusion criteria in the study, only normal or corrected to normal visual acuity participants were included. They could not have taken any medication or present any disease that could have influenced their cardiac functioning (e.g., tricyclic antidepressants or cardiac arrhythmia, respectively), or report any psychiatric or neurological diagnosis. The database is characterized by forty-seven right-handed, six left-handed, and two ambidextrous participants. Thirty-six participants completed secondary education, fifteen had a bachelor’s, and four had a master’s degree.

#### Signal Recording

**Figure 1:**
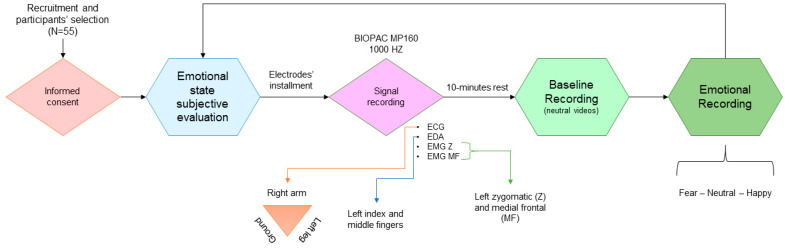
Data collection schema.

Figure 1 represents the process of signal recording. The cardiac signal (ECG), electrodermal activity (EDA), zygomatic (EMGZ), and medial frontalis (EMGMF) muscular information of each participant was recorded using BIOPAC MP160 data acquisition system and AcqKnowledge 5 software (BIOPAC Systems, Inc. in Goleta, CA, USA), with a sampling rate of 1000 Hz. Moreover, Ag/AgCl disposable vinyl electrodes (EL503; BIOPAC Systems, Inc.) and the corresponding conductive gel were used to acquire the signal, following the Lead II configuration [22], in ECG recording. EDA was collected at the Medial Phalanx of the index and middle fingers of the left hand. For EMG data collection, two electrodes were positioned on the zygomatic major muscle with a distance of 1 cm between each other; and two on the frontalis muscle, one at the center and the other with a distance of 1 cm and slightly towards the right side.

Before the task, the experimenter cleaned with ethyl alcohol (70%) the places where the electrodes would be placed. After placement, the participant waited at least 10 min before starting the experiment to allow the stabilization of the psychophysiological signals.

### 2.2. Signal Preprocessing

The participants were instructed to be as quite as possible during the signal acquisition. Nevertheless, the signals are always affected by noise. Therefore, to increase the signal quality, for the ECG, a Butterworth low pass filter with a cut-off frequency of 40 Hz was applied. For EMG, it was used a bandpass finite impulse response filter with 20 Hz and 450 Hz as cut-off frequencies. The EDA was filtered by means of a low pass Butterworth filter with 5 Hz cut-off frequency.

The level of physiological activation of participants is not the same across the 30-min movie duration. Moreover, some data segments may represent redundant data that may conduce to misleading results. Therefore, as a preliminary step, for each participant and each stimuli the information quantity of the signals was calculated, in a second base. To accomplish this step, the extended alphabet finite context model was used in each collected signal. An extended alphabet finite context model (xaFCM), estimates the probability of the next sequence of d > 0 symbols of the information source (depth-d) using the k > 0 immediate past symbols (order-k context). For each sequence of length k found, it counts the number of times each sequence of d symbols appeared right after it. The purpose was to use this model to give an approach for the number of bits that would be generated by a compressor [23].

The previous calculated information will give us a time series representing the amount of information during the 30 min experience. Our goal was to identify the 5-min interval with higher data quantity representation, to increase the information input on the classifier.

**Figure 2:**
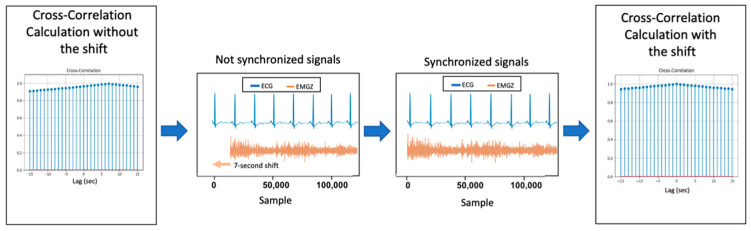
Process for time series synchronization.

Intuitively, the four collected signals should have the same behavior when information quantity is analyzed, since they depend on the participant’s response to the stimuli. Consequently, we decided to study the cross-correlation to evaluate the optimum lag that leads to the highest correlation value. In the optimal lag evaluation, it was observed that in most cases the optimum lag was zero. Nevertheless, some participants presented a lag different from 0. In case the optimal lag was equal to 0, we concluded that the time series were synchronized. On the other hand, in case optimal lag was different from 0, a shift corresponding to the module of the lag value was made, to the left in time if the optimal lag value was positive, and to the right if the optimum lag was negative, so the time series would be synchronized, as we can see in Figure 2. It is important to notice that the time shift was observed in few participants, and it always represents a small time shift. After this procedure, all signals became synchronized in terms of amount of information, which means that they are related to the experience segment that most activate the participant, minimizing redundant information, inside those 5-min window.

### 2.3. Feature Extraction and Selection

Feature extraction is the process of getting useful information from existing data. This is an important step, since the data available for classification are directly dependent on it. To extract all features from the signals, Neurokit was used. This library is a Python module that provides high-level integrative functions. From each signal, several features were extracted, as shown in Table 1. 

Notwithstanding, not all of them are relevant, so, in the next step, the ones that best represented the analyzed conditions were selected. To accomplish this step, we used the correlation threshold method, which found highly correlated features; one is selected in order to diminish data redundancy. The variance threshold was also used; the selected threshold was adapted to each feature space. This method selects only the features with high information quantity. After these two steps, the backward elimination method was used to select the features that minimizes the performance error. Table 2 presents the final selected features used for classification process.

**Figure 3:**
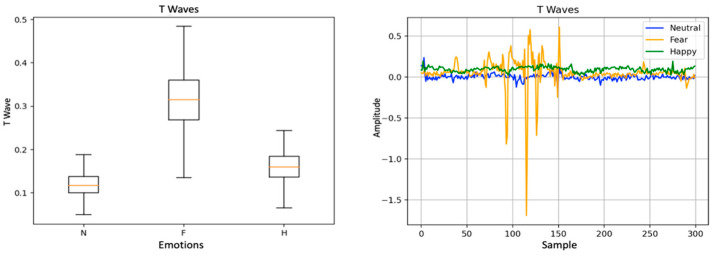
Representation of T Wave time series (at **right**), and the corresponding box plot (at **left**).

Figure 3 exemplifies one of the chosen features, the ECG T wave amplitude. By analyzing the box plot, we observed differences between the three conditions. Fear has associated an increased variability that can be associated with the sudden variation that can be observed in the time series (Figure 2, right chart). The remaining selected features, similarly, present a differentiation between the conditions.

### 2.4. Emotion Classification

The selected features were the input of two machine learning methods: random forest and neural networks (multilayer perceptron with backpropagation), two popular classifiers on emotion classification, given their theoretical characteristics. Random Forest is an algorithm constituted by several decision trees, therefore the results are the aggregation of the results obtained in each of the decision tree. This kind of methods are usually efficient in datasets with high variability. Neural networks are algorithms that find dependencies and relations between attributes, not available in a first analysis. Since there is not a physiological description of emotions, the relation between variables are not defined or known.

The classifiers’ parameters were found by grid search. In Random Forest, the used parameters for the classifiers with each isolated signal (ECG, Electromyogram zygomatic (EMGZ), Electromyogram medial frontalis (EMGMF) and EDA), and the combination of the two EMG signals were 8000 trees. When considering the classifier with all signals as input, 10,000 trees.

Considering the neural network, different structures were used depending on the input. Notwithstanding, the last layer is the output layer and has 3 nodes, that corresponds to the 3 emotions. Considering the neural network classifier with:

- ECG, EMGZ, and EDA signals as input, 4 layers were used. The first layer has 12 nodes. The second layer has 9 nodes and the third layer has 6 nodes;
- EMGMF as input, 3 layers were used. The first layer has 8 nodes. The second layer has 9 nodes;
- both EMG signals as input, 3 layers were used. The first layer has 10 nodes. The second layer has 24 nodes;
- all signals as input, 2 layers were used. The first layer has 28 nodes.

It is known that the physiological emotional response is not instantaneous, so there is a gap in time until the body reacts to the emotion. Moreover, the emotional information is encapsulated in the physiological signal, so the needed duration time for the methods to understand it is not known. Therefore, in this work, two frame durations were used and analyzed: 30 and 60 s.

Emotion recognition may be done as subject-independent (the data from a new subject is not known in the train dataset), and subject-dependent (the algorithm already knows the participant and its emotional response). In the two models, the data in the train and test dataset are different. To accomplish this goal, three approaches were planned. In the first approach, a division was made between the participants: 12 randomly selected out of 55 were used in the test dataset, and there is not any data from those participants on the train dataset. In the second division, again, 12 (out of 55) were used in test dataset. From the selected participants on the test dataset, one of the three emotions was randomly chosen to be part of the train dataset. The design of the test dataset was done in order to ensure that there is no unbalanced data for classification, both in train and test the same number of emotions were guaranteed. Again, the data on test dataset were not found in the training dataset. The third approach is similar to the second one; however, in this one, 30% of data were randomly chosen from each emotion from the participants in the test dataset and used in the training dataset, to provide more information of the participant to the model, leading to a subject-dependent analysis. In all three approaches, 10 iterations for random test dataset selection were performed to guarantee that the results are not biased to the sample. Furthermore, in the training phase, a shuffle split algorithm was used to split this dataset into a training and validation dataset. Shuffle split is a random permutation cross-validator, in this study, 20% was set for the validation set and 10 iteration folds were chosen.

The classification methods used were random forest and neural network, both reported in emotion identification literature. Notwithstanding, the literature is more focused on unimodal systems, using one physiological signal at a time. The performance metrics used to evaluate both models were:

- F1 Score: this metric conveys the balance between precision and recall. It gives a more accurate performance;
- sensitivity: it is the probability of the method classifying an emotion in a class, when it effectively belongs to it;
- specificity: it is the probability of the method not classifying an emotion in a specific class, when it does not belong to it.

## 3. Results

**Figure 4:**
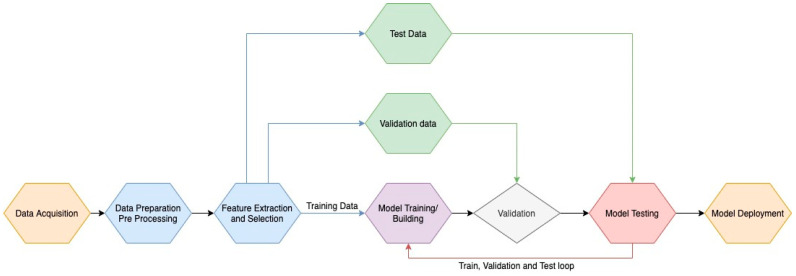
Multimodal emotion classification workflow.

Figure 4 represents the implemented process. Data are acquired from different physiological signals. Signals were filtered and the 5 min of maximum information window was selected on the data preparation and pre-processing block. Over the 5 min window, it was necessary to extract the features and then select the ones that better describe the physiological process. Data were then split in the training and test datasets. The test dataset was saved for later use, and the training dataset suffered another division in training and validation. Following, the model was trained, the validation dataset was used to validate, and the test dataset for testing. Ten iterations with the same follow-up were performed. Subsequently, the model results were analyzed and the model deployed.

For emotion recognition, two machine learning algorithms were used: the random forest and the neural network. In order to correctly compare the results from the two methods, the same training and testing datasets were used. In this context, the shuffle split with 10 iterations and a validation split with 20% were used. In Table 3, we present for both classifiers and for the two-frame duration the F1-score (corresponding to the best classifiers) obtained from the iterations of the shuffle split. Three conditions were tested:

(a) when there is no data sharing between train and test dataset (participants in one dataset are not in the other one);
(b) the data in training and testing is disjointly selected; nevertheless, one emotion (condition) of the participants in the test sample is in training and the other two conditions are in testing;
(c) from the participants in test sample, 30% of data in each condition were randomly selected and added to the train dataset.

In (a), we were evaluating a subject independent approach; in (b) and (c), a subject dependent approach was performed. Emotions are idiosyncratic, then it is expected that the algorithms need some participants and emotion response information to better describe the emotional context and response.

**Figure 5:**
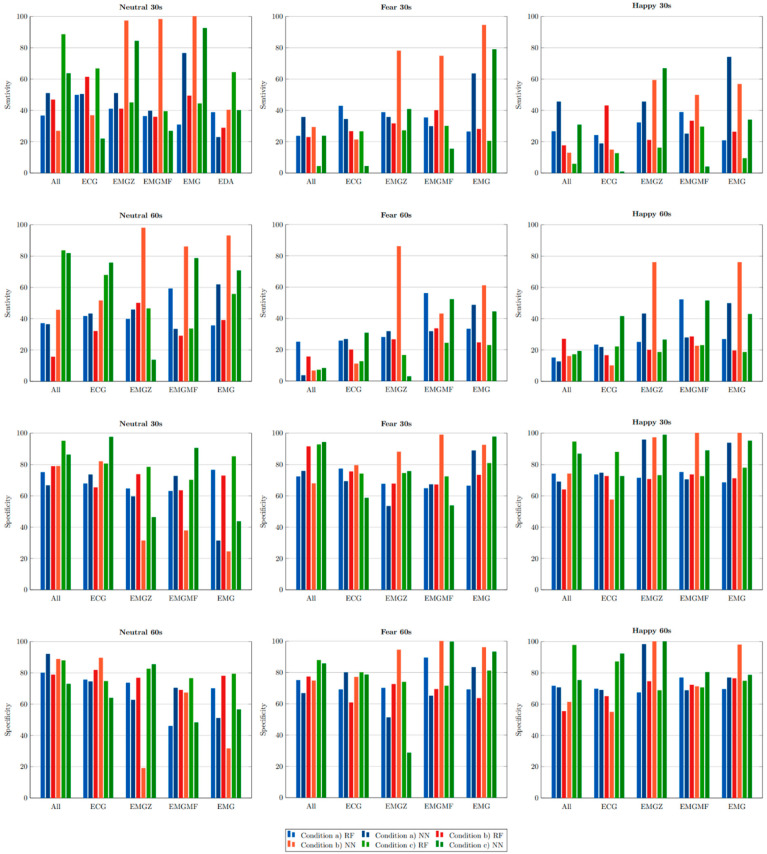
Sensitivity and specificity obtained for the two classifiers in different condition; (**a**) corresponds to the subject independent evaluation, (**b**) corresponds to the subject dependent evaluation, and (**c**) corresponds to emotion dependent evaluation.

In Figure 5, the sensitivity and specificity obtained in the classification (corresponding to the best classifiers) process are presented. In most of the cases, the classifier correctly predicts the classes, in exception of EMGZ and EMGMF. EMGZ better classifies happiness than fear and EMGMF better classifies fear than happiness.

Table 4 and Table 5 represent the global result of the mean accuracy of the 30s and 60s data frame (evaluating all splits). The result is presented as mean F1 score, standard deviation, maximum and minimum F1 score. Maximum and minimum are represented because the shuffle split is a random iteration model, and in some datasets, it is possible that it includes more information, and this leads to variation in final F1 score results.

## 4. Discussion

This study aimed to evaluate how well different signals described physiological response towards emotional stimuli, as well as what signal/combination of signals may better describe this response. To accomplish these aims, user independent and user dependent emotion recognition methods were presented. From the three approaches tested, the approach where 30% of the emotion was included in the train dataset had better results compared to the other two approaches. This supports the need of more information about the participant, since the emotion classification is dependent on the amount of information known by the algorithm.

In the case of the study between 30s and 60s, no conclusions can be drawn, since the shuffle split may be giving more useful information in one of the test cases. There is not a clear difference between the two-time frames. 

Considering the physiological representation/description of emotion, we observed that the best combination of signals for emotion description corresponds to use all the signals with all the selected features. In the case of 30s frame, with random forest, an accuracy of around 88% was obtained. In the case of 60s frame, with random forest, an accuracy of around 87% was obtained. With neural network, in the case of 30s frame an accuracy of 77% was obtained, and in the case of 60s frame, an accuracy of around 54% was obtained. Nevertheless, by analyzing the results, two signals (when considered isolated on classification) have obtained good results: ECG and EDA. When analyzing the sensitivity and specificity results, the ECG had more correct classifications than EDA. The major drawback of this signal is its level of noise, since a small movement of the hand will lead to high levels of noise. On the other hand, when collecting the ECG signal it is easy to hide the electrodes, although it is needed to use three electrodes.

The EMG also achieved good results when both collected signals (EMGZ and EMGMF) are combined. This occurs because the two separated signals will not provide the complete description of the emotional response, but together they complement their information and the emotional description is more accurate. The EMGZ captures more information at level of happiness, and the EMGMF captures more information at the level of fear. The good discriminative value of the EMG signals on the emotion stratification is in line with the research already done on facial expression evaluation, since the EMG signals were collected on the face. The electrode positioning on the face is more intrusive than the facial recognition. Nevertheless, the facial recognition needs a good focus of the camera on the face, which in real conditions is not always possible. Moreover, facing a camera is not always an easy task for some people. Therefore, the EMG can be a solution to monitor the facial reaction. Nevertheless, we are aware that this solution may only be viable on laboratory experiments, since real life data collection would need less intrusive and portable sensors.

Overall, in terms of machine learning algorithms, there are discrepancies between random forest and neural network in the three conditions described. In the case where the test includes participants who will not be known by the classifiers and in the case where the test includes an emotion from each participant, the neural network achieved better performance. In the case where 30% of the emotions were supplied to the training dataset, the random forest got better performance. Therefore, the results indicate that the neural network better describes the emotional context when a subject or emotion independent test is performed, while the random forest better describes the subject and emotion dependent context.

It is also important to notice that we are using the information interval with higher information quantity, which may not be observed in all data collection protocols and, also, not during all the experiment. Nevertheless, to identify each signal contribution to emotion recognition is a truly important step, in order to guarantee equal initial conditions for all the evaluated signals.

Knowledge about emotional response, and particularly, the possibility to obtain accurate, non-redundant and objective emotional data using automatic systems for emotion identification and modelling has important implications. For instance, this system would be crucial in situations where a real setup must be planned and where the ecological validity is very important. Moreover, it is important to know which consequences stem from selecting one signal in detriment of another.

Our results suggest that the combination of more than one signal would produce better results in emotion classification; however, it may not be always possible. Frequently, due to financial, material, or methodological constraints, it is not possible to measure multiple signals at a time. Therefore, knowing how informative a signal or a combination of signals may be, helps to design more cost-effective set-ups. This study provides important guidelines about which signals are more informative for the classification of fear, happiness, and neutral emotion, as well as about possible errors associated with situations where only one of the mentioned signals was used.

Finally, this study yields important applications. For instance, in the context of mental health, where frequently more objective data about the emotional response of the patients is lacking, having a system able to detect, process, and interpret physiological responses associated with the emotional state would be an added value to the diagnosis, monitoring and intervention process. Furthermore, these systems can be integrated and used to develop cost-effective, non-invasive, and portable devices with the potential to foster an efficient monitoring of emotional responses, emotion regulation strategies and communication with caregivers and health agents. Apart from the mental health context, these systems can be used in other contexts, including to monitor disorders associated with somatic alterations or even populations with emotional processing and communication difficulties, such as autism spectrum disorders [24], as well as in situations where this information can be highly informative about an individual’s cognition and behavior (e.g., on the monitoring of long-distance motorists). 

## 5. Conclusions

This research analyzed the physiological component of emotion in different emotional conditions (fear, happiness and neutral), using automatic systems for emotion identification. Our results suggest that the ECG signal seems to be the most informative in emotion stratification. The use of facial EMG in emotion is dependent on monitoring two (or more) muscles, allowing to identify facial expression changes by corresponding muscular contractions. Nevertheless, if all signals are used on emotion identification, a higher accuracy is achieved, since all signals are representative of different information. This physiological model of emotions has important research and clinical implications, by providing valuable information about the value and weight of physiological signals for emotional classification, which can critically drive effective evaluation, monitoring, and intervention regarding emotional processing and regulation, considering multiple contexts.

## Figures and Tables

**Table 1:** Extracted features from collected biosignals: electrocardiogram (ECG), electromyogram (EMG), electrodermal activity (EDA).

Signal	Features Extracted
**ECG**	R Peaks, Cardiac Cycles, T Waves, P Waves, Q Waves, Cardiac Cycles Signal Quality, Average Signal Quality, ECG Signal Quality, ECG Raw, ECG Filtered, Heart Rate, ECG Systole, RR Interval, Heart Rate Variability high frequency (ECG HRV HF), Heart Rate Variability low frequency (ECG HRV LF), Heart Rate Variability Ultra Low Frequency (ECG HRV ULF), Heart Rate Variability Very High Frequency (ECG HRV VHF), Detrended Fluctuation Analysis (DFA) 1, Detrended Fluctuation Analysis 2, Shannon, Sample Entropy, Heart Rate Variability Very Low Frequency (ECG HRV VLF), Electrocardiographic Artifacts, Root Mean Square of Successive Differences (RMSSD), mean of distance between Normal to Normal peaks (meanNN), standard deviation of distance between Normal to Normal peaks (sdNN), coefficient of variation of distance between Normal to Normal peaks (cvNN), coefficient of variation of sdNN(CVSD), median of distance between Normal to Normal peaks (medianNN), mean absolute deviation of distance between Normal to Normal peaks (madNN), Median-based Coefficient of Variation (mcvNN), percentage of NN intervals differing by more than 50 ms (pNN50), pNN20, Entropy Multiscale Area Under the Curve (AUC), Entropy SVD, Entropy Spectral VLF, Triang, Shannon h, Ultra Low Frequency (ULF), Very Low Frequency (VLF), Low Frequency (LF), High Frequency (HF), Very High Frequency (VHF), Correlation Dimension, Entropy Spectral LF, Entropy Spectral HF, Fisher Info, FD Petrosian, Total Power, LFn, HFn, LF/HF, LF/P, HF/P, FD Higushi
**EDA**	EDA Raw, EDA Filtered, EDA Phasic, EDA Tonic, Skin Conductance Response (SCR) Recoveries, SCR Peaks, SCR Recovery Indexes, SCR Peaks Amplitudes, SCR Onsets, SCR Peaks Indexes
**EMG**	EMG Raw, EMG Filtered, EMG Envelope, EMG Activation, EMG Pulse Onsets

**Table 2:** Selected features for emotional classification, from electrocardiogram (ECG), electrodermal activity (EDA), and electromyogram (EMG).

Signal	Selected Feature
**ECG**	Heart Rate, ECG RR Intervals, Heart Rate Variability High Frequency, Heart Rate Variability Low Frequency, Heart Rate Variability Ultra Low Frequency, T Waves
**EDA**	EDA Tonic, Skin Conductance Response (SCR) Peaks Indexes
**EMG**	EMG Envelope, EMG Pulse Onsets

**Table 3:** F1-score obtained for the two classifiers; (a) corresponds to the subject independent evaluation, (b) corresponds to the subject dependent evaluation, and (c) corresponds to emotion dependent evaluation.

		Random Forest (%)						Neural Network (%)					
Emotion	Condition	Neutral		Fear		Happy		Neutral		Fear		Happy	
		30s	60s	30s	60s	30s	60s	30s	60s	30s	60s	30s	60s
**All**	**(a)**	39	42	53	53	49	54	50	48	43	59	39	48
	**(b)**	50	24	50	40	52	45	32	54	52	49	52	33
	**(c)**	89	80	89	85	86	82	78	58	82	57	73	47
**ECG**	**(a)**	46	44	27	38	44	48	49	44	38	45	50	49
	**(b)**	53	38	42	36	27	51	43	60	30	29	61	50
	**(c)**	71	62	57	61	63	60	34	61	56	61	68	44
**EMGZ**	**(a)**	39	41	32	37	41	45	44	41	46	50	17	23
	**(b)**	47	51	49	47	48	51	58	54	25	22	29	26
	**(c)**	48	51	48	47	56	55	58	19	56	57	1	29
**EMGMF**	**(a)**	34	35	40	36	35	39	41	44	38	32	44	41
	**(b)**	44	30	39	46	41	42	61	68	40	17	66	65
	**(c)**	39	37	46	49	44	47	35	56	55	41	80	50
**EMG**	**(a)**	35	36	42	38	46	43	49	48	43	32	28	38
	**(b)**	44	43	46	48	45	49	57	56	9	51	44	34
	**(c)**	51	57	63	52	71	61	61	55	26	47	74	46
**EDA**	**(a)**	42	38	42	49	46	35	29	40	51	62	47	33
	**(b)**	46	34	48	47	47	44	47	57	67	64	83	77
	**(c)**	61	52	61	56	52	54	53	48	73	63	60	63

**Table 4:** Global result for 30s frame. The mean (standard deviation) [Maximum Minimum] F1 score obtained for the 30s classifiers in each iteration; (a) corresponds to the subject independent evaluation, (b) corresponds to the subject dependent evaluation, and (c) corresponds to emotion dependent evaluation.

Signal	Condition	Random Forest (Mean % ± std %)	Neural Network (Mean % ± std %)
**All**	**(a)**	39.07 (1.61) [Max: 43.64%, Min: 37.94%]	35.82 (1.05) [Max: 38.32%, Min: 34.37%]
	**(b)**	30.93 (1.40) [Max: 35.47%, Min: 29.94%]	31.00 (3.02) [Max: 31.74%, Min: 29.53%]
	**(c)**	76.25 (1.42) [Max: 77.70%, Min: 73.13%]	50.16 (3.41) [Max: 52.11%, Min: 40.14%]
**ECG**	**(a)**	36 (0.78) [Max: 36.73%, Min: 34.25%]	37 (0.77) [Max: 38.87%, Min: 36.10%]
	**(b)**	32.01 (0.83) [Max: 33.21% Min: 30.31%]	34.63 (1.16) [Max: 35.18% Min: 30.11%]
	**(c)**	49.79 (3.35) [Max: 58.10%, Min: 46.62%]	37.43 (0.86) [Max: 38.24%, Min: 35.47%]
**EMGZ**	**(a)**	34.23 (0.20) [Max: 34.53%, Min: 33.82%]	33.75 (1.05) [Max: 38.30%, Min: 34.87%]
	**(b)**	31.60 (0.96) [Max: 32.74% Min: 30.08%]	31.12(0.88) [Max: 34.01% Min: 30.97%]
	**(c)**	38.33 (1.66) [Max: 39.80%, Min: 34.95%]	36.69 (1.40) [Max: 39.70%, Min: 34.69%]
**EMGMF**	**(a)**	33.82 (0.29) [Max: 34.33%, Min: 33.32%]	33 (0.27) [Max: 34.41%, Min: 33.46%]
	**(b)**	32.73 (0.43) [Max: 33.37%, Min: 31.68%]	35.90 (2.88) [Max: 39.97%, Min: 29.48%]
	**(c)**	39.46 (1.16) [Max: 41.65%, Min: 38.17%]	41.18 (5.23) [Max: 51.98%, Min: 37.05%]
**EMG**	**(a)**	35.80 (0.43) [Max: 36.33%, Min: 35.03%]	36.00 (0.63) [Max: 37.04%, Min: 35.20%]
	**(b)**	35.28 (1.08) [Max: 36.73% Min: 33.53%]	27.67 (1.49) [Max: 29.49% Min: 24.85%]
	**(c)**	49.01 (1.62) [Max: 51.26%, Min: 45.90%]	36.02 (2.08) [Max: 37.85%, Min: 30.04%]
**EDA**	**(a)**	37.53 (1.32) [Max: 38.78%, Min: 34.25%]	38.98 (0.64) [Max: 39.90%, Min: 37.82%]
	**(b)**	36.23 (1.59) [Max: 40.12% Min: 34.93%]	42.32 (4.46) [Max: 53.90% Min: 39.03%]
	**(c)**	48.79 (2.13) [Max: 51.72%, Min: 43.11%]	42.77 (3.40) [Max: 46.75%, Min: 33.82%]

**Table 5:** Global results for 60s frame. The mean (standard deviation) [Maximum Minimum] F1 score obtained for the 60s classifiers in each iteration; (a) corresponds to the subject independent evaluation, (b) corresponds to the subject dependent evaluation, and (c) corresponds to emotion dependent evaluation.

Signal	Condition	Random Forest (Mean % ± std %)	Neural Network (Mean % ± std %)
**All**	**(a)**	41.71 (1.26) [Max: 44.57% Min: 39.95%]	43.69 (0.95) [Max: 45.41%, Min: 42.65%]
	**(b)**	26.80 (1.68) [Max: 30.21%, Min: 24.28%]	28.69 (1.83) [Max: 31.28%, Min: 23.68%]
	**(c)**	73.37 (3.72) [Max: 80.55%, Min: 67.55%]	38.68 (1.97) [Max: 43.64%, Min: 36.25%]
**ECG**	**(a)**	38.68 (0.80) [Max: 40.42%, Min: 37.71%]	38.95 (0.88) [Max: 41.38%, Min: 38.32%]
	**(b)**	28.88 (1.76) [Max: 31.67%, Min: 25.63%]	32.58 (2.36) [Max: 34.28%, Min: 26.32%]
	**(c)**	52.15 (2.16) [Max: 55.62%, Min: 48.38%]	40.80 (2.04) [Max: 43.77%, Min: 38.15%]
**EMGZ**	**(a)**	35.19 (0.40) [Max: 35.68%, Min: 34.40%]	34.97 (0.41) [Max: 35.58%, Min: 34.11%]
	**(b)**	36.06 (0.65) [Max: 37.88%, Min: 35.83%]	34.93 (0.80) [Max: 35.61%, Min: 32.77%]
	**(c)**	41.32 (0.77) [Max: 42.55%, Min: 39.92%]	35.21 (0.55) [Max: 36.71%, Min: 34.69%]
**EMGMF**	**(a)**	33.13 (0.31) [Max: 33.69%, Min: 32.70%]	33.88 (0.39) [Max: 34.42%, Min: 33.10%]
	**(b)**	32.52 (1.32) [Max: 35.85%, Min: 30.58%]	34.78 (1.97) [Max: 36.93%, Min: 29.62%]
	**(c)**	36.23 (1.62) [Max: 37.50%, Min: 31.67%]	34.25 (2.19) [Max: 36.40%, Min: 30.05%]
**EMG**	**(a)**	36.15 (0.59) [Max: 37.65%, Min: 35.61%]	34.50 (0.59) [Max: 35.37%, Min: 33.37%]
	**(b)**	32.13 (3.02) [Max: 35.55%, Min: 25.85%]	25.32 (3.46) [Max: 29.40%, Min: 19.53%]
	**(c)**	48.24 (1.88) [Max: 52.05%, Min: 46.13%]	37.32 (2.09) [Max: 40.76%, Min: 34.10%]
**EDA**	**(a)**	37.02 (0.66) [Max: 37.89%, Min: 36.09%]	37.93 (0.98) [Max: 39.52%, Min: 36.79%]
	**(b)**	37.08 (0.52) [Max: 38.03%, Min: 36.35%]	40.73 (1.75) [Max: 44.60%, Min: 38.37%]
	**(c)**	41.66 (1.56) [Max: 44.55%, Min: 38.67%]	37.61 (1.65) [Max: 41.48%, Min: 35.36%]

## References

[1] Scherer K.R. (2005). What are emotions? And how can they be measured?. Soc. Sci. Inf..

[2] Dolan R.J. (2002). Emotion, cognition, and behavior. Science.

[3] Tooby J., Cosmides L., Lewis M., Haviland-Jones J.M., Barrett L.F. (2008). The evolutionary psychology of the emotions and their relationship to internal regulatory variables. Handbook of Emotions.

[4] DeSteno D., Gross J.J., Kubzansky L. (2013). Affective science and health: The importance of emotion and emotion regulation. Heal. Psychol..

[5] Consedine N.S., Moskowitz J.T. (2007). The role of discrete emotions in health outcomes: A critical review. Appl. Prev. Psychol..

[6] Mauss I.B., Robinson M.D. (2009). Measures of emotion: A review. Cogn. Emot..

[7] Calvo R., D’Mello S. (2010). Affect detection: An interdisciplinary review of models, methods, and their applications. Affect. Comput. IEEE Trans..

[8] Shu L., Yu Y., Chen W., Hua H., Li Q., Jin J., Xu X. (2020). Wearable Emotion Recognition Using Heart Rate Data from a Smart Bracelet. Sensors.

[9] Dzedzickis A., Kaklauskas A., Bucinskas V. (2020). Human emotion recognition: Review of sensors and methods. Sensors.

[10] Romeo L., Cavallo A., Pepa L., Berthouze N., Pontil M. (2019). Multiple instance learning for emotion recognition using physiological signals. IEEE Trans. Affect. Comput..

[11] Cacioppo J.T., Tassinary L.G., Berntson G. (2007). Handbook of Psychophysiology.

[12] Ferreira J., Brás S., Silva C.F., Soares S.C. (2017). An automatic classifier of emotions built from entropy of noise. Psychophysiology.

[13] Brás S., Ferreira J.H.T., Soares S.C., Pinho A.J. (2018). Biometric and Emotion Identification: An ECG Compression Based Method. Front. Psychol..

[14] Cai J., Liu G., Hao M. The research on emotion recognition from ECG signal. Proceedings of the 2009 International Conference on Information Technology and Computer Science.

[15] Bird J.J., Manso L.J., Ribeiro E.P., Ekart A., Faria D.R. A Study on Mental State Classification using EEG-based Brain-Machine Interface. Proceedings of the 9th International Conference on Intelligent Systems 2018: Theory, Research and Innovation in Applications.

[16] Rigas G., Katsis C.D., Ganiatsas G., Fotiadis D.I. (2007). A user independent, biosignal based, emotion recognition method. Lect. Notes Comput. Sci..

[17] Mokhayeri F., Akbarzadeh-T M.R., Toosizadeh S. Mental stress detection using physiological signals based on soft computing techniques. Proceedings of the 2011 18th Iran. Conf. Biomed. Eng. ICBME 2011.

[18] Gouizi K., Reguig F.B., Maaoui C. Analysis physiological signals for emotion recognition. Proceedings of the 7th International Workshop on Systems, Signal Processing and their Applications.

[19] James S.L., Abate D., Abate K.H., Abay S.M., Abbafati C., Abbasi N., Abdollahpour I. (2018). Global, regional, and national incidence, prevalence, and years lived with disability for 354 diseases and injuries for 195 countries and territories, 1990–2017: A systematic analysis for the Global Burden of Disease Study 2017. Lancet.

[20] Wang C., Pan R., Wan X., Tan Y., Xu L., Ho C.S., Ho R.C. (2020). Immediate psychological responses and associated factors during the initial stage of the 2019 Coronavirus Disease (COVID-19) epidemic among the general population in China. Int. J. Environ. Res. Public Health.

[21] Torales J., O’Higgins M., Castaldelli-Maia J.M., Ventriglio A. (2020). The outbreak of COVID-19 coronavirus and its impact on global mental health. Int. J. Soc. Psychiatry.

[22] Berntson Q.K.S., Lozano D. (2007). Cardiovascular Psychophysiology.

[23] Carvalho J.M., Brás S., Pratas D., Ferreira J., Soares S.C., Pinho A.J. (2018). Extended-alphabet finite-context models. Pattern Recognit. Lett..

[24] Torrado J.C., Gomez J., Montoro G. (2017). Emotional self-regulation of individuals with autism spectrum disorders: Smartwatches for monitoring and interaction. Sensors.

